# Potential Impact of Targeted HIV Pre-Exposure Prophylaxis Uptake Among Male Sex Workers

**DOI:** 10.1038/s41598-020-62694-5

**Published:** 2020-03-27

**Authors:** William C. Goedel, Matthew J. Mimiaga, Maximilian R. F. King, Steven A. Safren, Kenneth H. Mayer, Philip A. Chan, Brandon D. L. Marshall, Katie B. Biello

**Affiliations:** 1 Department of Epidemiology, School of Public Health, Brown University, Providence, Rhode Island United States; 2 Department of Behavioral and Social Sciences, School of Public Health, Brown University, Providence, Rhode Island United States; 3 Fenway Institute, Fenway Health, Boston, Massachusetts United States; 4 Department of Psychology, College of Arts and Sciences, University of Miami, Coral Gables, Florida United States; 5 Division of Infectious Diseases, Beth Israel Deaconess Medical Center, Boston, Massachusetts United States; 6 Department of Global Health and Population, T.H. Chan School of Public Health, Harvard University, Cambridge, Massachusetts United States; 7 Department of Medicine, Warren Alpert Medical School, Brown University, Providence, Rhode Island United States

**Keywords:** HIV infections, Epidemiology

## Abstract

Little is known about the potential population-level impact of HIV pre-exposure prophylaxis (PrEP) use among cisgender male sex workers (MSWs), a high-risk subset of cisgender men who have sex with men (MSM). Using an agent-based model, we simulated HIV transmission among cisgender MSM in Rhode Island to determine the impacts of PrEP implementation where cisgender MSWs were equally (“standard expansion”) or five times as likely (“focused expansion”) to initiate PrEP compared to other cisgender MSM. Without PrEP, the model predicted 920 new HIV infections over a decade, or an average incidence of 0.39 per 100 person-years. In a focused expansion scenario where 15% of at-risk cisgender MSM used PrEP, the total number of new HIV infections was reduced by 58.1% at a cost of $57,180 per quality-adjusted life-year (QALY) gained. Focused expansion of PrEP use among cisgender MSWs may be an efficient and cost-effective strategy for reducing HIV incidence in the broader population of cisgender MSM.

## Introduction

Cisgender male sex workers, generally defined as cisgender men who have sex with men (MSM) in exchange for money, drugs, or other goods, have significantly elevated HIV prevalence compared to the general population of other cisgender MSM^1,2^. Several behavioral factors associated with increased risk of HIV infection have been reported among cisgender male sex workers, including a high number of sexual partners, more frequent engagement in condomless anal intercourse, and injection and non-injection drug use^1,2^. These behavioral risks are exacerbated by the illegal nature of sex work in many settings, which contributes to stigma, harassment, violence, and low health care engagement in this population^1^.

Given the intersecting nature of structural vulnerabilities and elevated risk of HIV infection among some populations of cisgender male sex workers^1^, as well as diversity of sexual identities and behaviors present in this population, they may have unique HIV prevention needs. Interventions that can be controlled by cisgender male sex workers themselves are critical. Condom use may be difficult for several reasons. It may be difficult to negotiate condom use with clients in circumstances where the client offers additional money to not use one, particularly when men are using drugs or experiencing economic hardship^1^. Further, in many jurisdictions, police often use confiscation of multiple condoms as evidence to support prostitution-related criminal charges, making men reluctant to carry them^1^. As such, pre-exposure prophylaxis (PrEP) may represent an acceptable and effective HIV prevention method for cisgender male sex workers^3–6^.

Previous studies using mathematical models have shown that expanded PrEP use can reduce HIV incidence among cisgender MSM and that focused PrEP uptake among high-risk cisgender MSM may increase the efficiency and cost-effectiveness of PrEP implementation^7–10^. However, little is known about the potential impact of increasing PrEP uptake among cisgender male sex workers. To this end, we adapted an existing agent-based model of HIV transmission among cisgender MSM in Rhode Island^11^, and parameterized the model using data from a recent assessment of the networks of cisgender male sex workers in New England^12^, to identify the potential impacts of focused PrEP expansion in this population.

## Methods

### Model setting

In an agent-based model, an epidemic system is modeled as a collection of individual decision-making entities referred to as agents^13^. Through stochastic processes, agents make decisions on the basis of a set of rules and execute various behaviors appropriate for the system they represent^13^, such as forming sexual partnerships and engaging in sexual behavior. Population-level properties emerge as a result of the behavior and interactions of these agents, allowing for an understanding of how changes in individual behavior (such as PrEP use) impact population-level outcomes (like HIV incidence)^13^.

Our model, written and executed in Python (Version 3.7.4), simulated HIV transmission for ten years within a population of 25,000 individuals, representing all adult cisgender MSM in Rhode Island^14^. Rhode Island represents a unique site to perform the study given the abundance of local data among a statewide population. The state also had laws which facilitated sex work for many years and also hosts the only dedicated community-based organization focused on male sex workers in the country^15^.

This agent-based model simulated a population in steady state, where individuals left the population at death or due to aging out at 65 years old. The model progressed in a series of discrete time-steps, each representing one calendar month. Detailed information on parameter values, calibration, key assumptions, and data sources are shown in the Supplemental Appendix. Parameters are informed by data from Rhode Island, wherever possible, as well as by estimates from the existing literature as needed.

### Sexual behavior and sexual network formation

Agents were assigned one of three classes at model initialization: cisgender male sex workers (representing about 4% of all agents), potential clients of cisgender male sex workers (representing about 5% of all agents), and all other cisgender MSM (representing about 91% of the all agents). Parameters governing the behavior of cisgender male sex workers were drawn from distributions specific to this agent class, while parameters governing the behavior of all other cisgender MSM were drawn from separate distributions.

Upon agent creation, each agent has a “desired” annual partner number drawn from a negative binomial distribution matching their agent characteristics. This number serves as the target mean number of partners per year within a standard normal distribution for the agent’s lifespan in the model. Every year, the target partner number for the agent is pulled from this distribution. This process allows agents to have implicit preferences in partner acquisition patterns while exhibiting variability in their behaviors from year to year. During each one-month time-step, agents searched for and acquired partners and engaged in sex acts, where the number of sex acts within a given partnership was assigned as an average between each agent’s desired number of sex acts per partner per month (with target mean numbers of sex acts per partner per month drawn from a Poisson distribution at model initialization through a similar process). Further, the probability of condom use during a given sex act is a function of the number of prior contacts between two agents, where condom use is less likely in partnerships with higher numbers of prior contacts. All new partnerships were assigned a duration at formation drawn from a distribution informed by empirical data, allowing partnerships to dissolve to create dynamic sexual networks as the model progressed.

### HIV transmission, treatment, and progression

The base per-act probabilities of transmission associated with all sexual risk behaviors were derived from a recent meta-analysis^16^. To reduce computational costs, only behaviors occurring within serodiscordant dyads were simulated explicitly. All HIV-infected individuals experienced a monthly probability of progression to AIDS based on their use of antiretroviral treatment and achievement of viral suppression.

At model initialization, we assumed that 97% of all cisgender male sex workers and 89% of all other cisgender MSM had ever been tested for HIV in their lifetime, with a monthly probability of testing of 7% and 5%, respectively (assuming that 84% of cisgender male sex workers and 62% of all other cisgender MSM are tested within a twelve-month period). It is assumed that 64% of cisgender male sex workers living with HIV infection and 55% of all other cisgender MSM living with HIV infection were using antiretroviral treatment at any point and that 60% of cisgender male sex workers and 82% of all other cisgender MSM using antiretroviral treatment had achieved viral suppression^17^. Upon initiating treatment, the base probabilities of transmission were reduced by 96% among individuals with viral suppression and by 17% among individuals without viral suppression^16^. In the base case scenario, where PrEP is not available, condom use and these reduced probabilities of transmission with the provision of antiretroviral treatment represent the only interventions taken to reduce HIV transmission.

### Pre-exposure prophylaxis implementation

The target PrEP coverage level for a given scenario was imposed immediately upon implementation in the model and maintained throughout the simulation (Supplemental Fig. 1). During each time-step, a pre-determined number of prescriptions were made available. Agents who dropped out of the PrEP program were replaced by new agents. In all scenarios, agents who use PrEP could achieve optimal (i.e., take four or more doses per week, resulting in a 96% decrease in the per-act probability of HIV acquisition) or suboptimal adherence (i.e., take one to three doses per week, resulting in a 76% decrease in the per-act probability of HIV acquisition) with probabilities informed by observed rates of self-reported adherence among cisgender MSM in the statewide PrEP program in Rhode Island^18^. Agents who use PrEP were also subject to a monthly probability of discontinuation, informed by observed rates of retention in clinical care among cisgender MSM in the PrEP program^18^. It is assumed that agents engage in similar sexual behaviors when using and not using PrEP (i.e., there is no behavior change following the discontinuation of PrEP use).

### Cost and utility assumptions

The annual operating cost of providing PrEP to cisgender MSM living in Rhode Island was estimated using a health system perspective. Patient monitoring costs, including those associated with staff time, were derived from a cost analysis of the PrEP program in Rhode Island^19^. Although not all patients present for quarterly visits or receive all laboratory tests per clinical recommendations, we assumed optimal implementation of these recommendations^20^. As such, our estimates represent the upper bound of the costs of PrEP implementation. All cost and utility assumptions are shown in Supplemental Appendix^19,21,22^. All costs are expressed in 2016 United States dollars and assume 3% discounting.

### Model calibration

The model was calibrated using Latin hypercube sampling to reproduce the observed trends in the number of cisgender MSM living with diagnosed HIV infection in Rhode Island between 2008 and 2014 (Supplemental Fig. 2)^23^. After calibration, each model run incorporated a burn-in period of 36 months to recreate these trends, after which PrEP was introduced and its effects on HIV transmission were observed for ten years.

### Model scenarios

The model simulated HIV transmission over ten years. In the base case scenario, transmission is simulated without PrEP implementation. In six counterfactual scenarios, different populations of cisgender MSM were engaged with PrEP services over a decade. In the model, agents could only initiate PrEP if they met current prescribing recommendations^20^. PrEP uptake was implemented in line with current recommendations to achieve one of three target coverage levels (15%, 20%, or 25% of all HIV-uninfected MSM). In three of these six scenarios (hereby referred to as “standard” uptake), PrEP uptake was not focused beyond these recommendations. In the remaining scenarios, PrEP expansion is further focused such that eligible male sex workers were five times as likely to initiate PrEP as all other cisgender MSM (hereby referred to as “focused” uptake). To permit maximum comparability between scenarios, overall coverage was held constant over time. Each model scenario is simulated for 1,000 unique iterations.

### Outcome measures: Epidemiologic impact of pre-exposure prophylaxis implementation

The primary outcome measures were the annual HIV incidence rate (expressed as the number of new HIV infections per 100 person-years) and the cumulative number of HIV infections over ten years. In addition, we report the number of HIV infections averted and the associated percent reduction in HIV incidence relative to the base case scenario where no individuals use PrEP. All measures were reported for the overall population and separately for cisgender male sex workers, their cisgender male clients, and all other cisgender MSM in the population. All outcome measures are presented with 95% simulation intervals (SIs).

### Outcome measures: Economic impact of pre-exposure prophylaxis implementation

As a measure of the economic impact and benefits of PrEP implementation, we calculated incremental cost-effectiveness ratios (ICERs), expressed as the cost per QALY gained^24^. A scenario is considered cost-effective if the intervention cost was less than $100,000 per QALY gained^24^.

### Sensitivity analyses

Sensitivity analyses were conducted to examine the robustness of the primary analyses to uncertain model parameters that might impact the observed effect of PrEP implementation, including the difference in the probabilities of optimal adherence and persistence among cisgender male sex workers relative to all other cisgender MSM, where cisgender male sex workers were 25% less likely to be achieve optimal adherence in a given time-step and 25% more likely to discontinue PrEP use in a given time-step.

## Results

**Figure 1:**
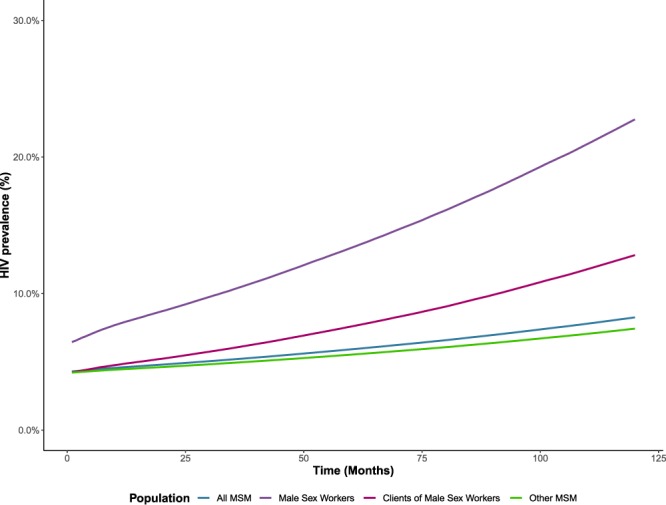
Trends in the prevalence of HIV infections from January 2015 to December 2024 among (**a**) all gay, bisexual, and other cisgender men who have sex with men (MSM); (**b**) cisgender male sex workers; (**c**) clients of cisgender male sex workers; and (**d**) all other cisgender MSM in a scenario with no PrEP implementation.

In the absence of PrEP implementation, the model predicted that HIV prevalence would increase from 4.3% (95% SI: 4.0–4.6%) in January 2015 to 8.3% (7.5–9.0%) in December 2024 in the full population. These increases in HIV prevalence were evident across all subgroups (Fig. 1), including male sex workers (from 6.4% to 22.8%), their clients (from 4.2% to 12.8%), and all other MSM (from 4.2% to 7.4%). The model predicted an average of 92 new HIV infections per year (95% SI: 52–146), corresponding to an annual incidence rate of 0.39 infections per 100 person-years (95% SI: 0.22–0.63). Although the majority of new infections occurred among other MSM (67; 95% SI: 37–108), annual incidence rates were highest among male sex workers (1.89; 95% SI: 0.72–3.47) and their clients (0.92; 95% SI: 0.26 to 1.84).

### Epidemiologic impact of pre-exposure prophylaxis implementation

**Figure 2:**
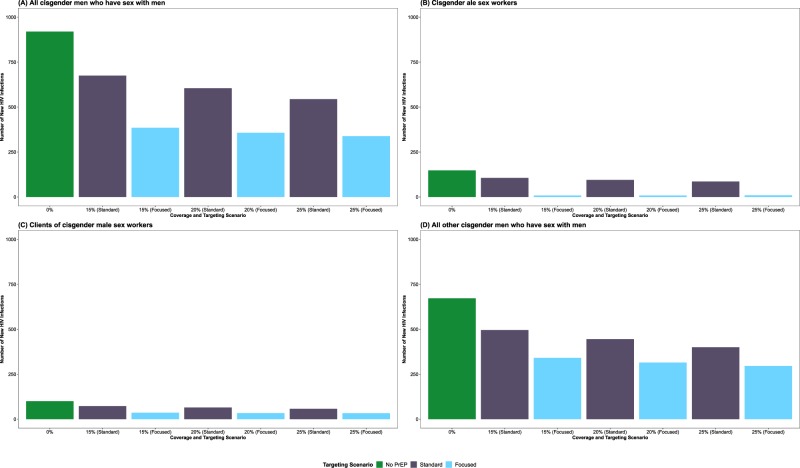
Number of incident HIV infections between January 2015 and December 2024 among (**A**) all gay, bisexual, and other cisgender men who have sex with men (MSM); (**B**) cisgender male sex workers; (**C**) cisgender clients of male sex workers; and (**D**) all other cisgender MSM by pre-exposure prophylaxis (PrEP) coverage level (15%, 20%, and 25%) and targeting scenario (standard and focused expansion).

Among all MSM, the total number of new HIV infections was reduced in both the standard and focused uptake scenarios (Fig. 2, Panel A). With 15% of eligible MSM using PrEP for ten years in a standard uptake scenario, the number of new HIV infections was reduced by 34.5% (95% SI: 21.5–46.6%), representing 317 infections averted (95% SI: 198–429) over the ten-year simulation period. With equivalent coverage in a focused expansion uptake scenario, the number of new HIV infections was reduced by 58.1% (95% SI: 50.2–65.1%) overall, representing 534 infections averted (95% SI: 462–599).

Among male sex workers, the total number of new HIV infections was reduced in both the standard and focused uptake scenarios (Fig. 2, Panel B), with substantial reductions observed in the focused uptake scenarios. With 15% of eligible MSM using PrEP for ten years in a standard uptake scenario, the number of new HIV infections was reduced by 28.5% (95% SI: 7.4–45.9%) among male sex workers, representing 42 infections averted (95% SI: 11–67). In a targeted uptake scenario with equivalent coverage, the number of new HIV infections was reduced by 94.4% (95% SI: 89.9–98.0%), representing 140 infections averted among male sex workers (95% SI: 133–145). Focused expansion also increased the number of averted HIV infections among their clients (Fig. 2, Panel C) and all other MSM (Fig. 2, Panel D).

**Figure 3:**
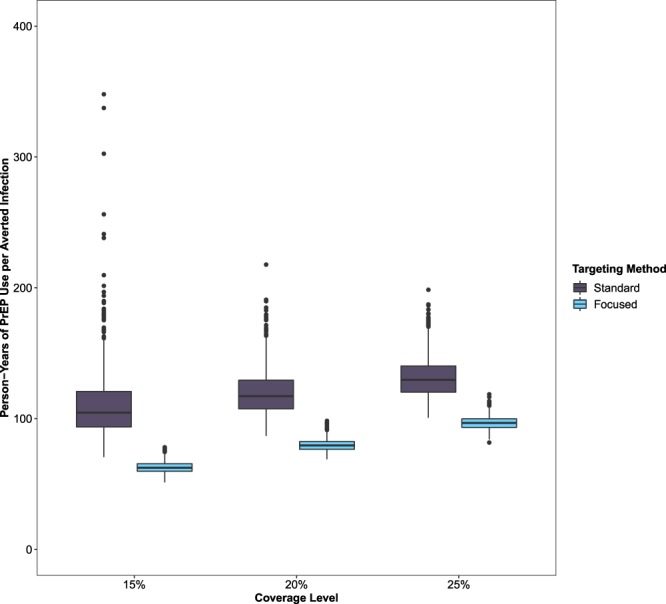
Person-years of pre-exposure prophylaxis (PrEP) use per averted HIV infection among gay, bisexual, and other cisgender men who have sex with men (MSM) in Rhode Island by coverage level (15%, 20%, or 25%) and targeting scenario (standard uptake and focused expansion).

Despite increases in the number of HIV infections averted with increasing numbers of individuals using PrEP, the efficiency of PrEP use at the population level decreased with increasing coverage (Fig. 3), from 110 person-years of PrEP use per HIV infection averted (95% SI: 78–168) with 15% of eligible MSM using PrEP for ten years to 131 person-years of PrEP use per HIV infection averted (95% SI: 108–166) with 25% of eligible MSM using PrEP for ten years in standard uptake scenarios. However, relative to these standard uptake scenarios, focused uptake improved the efficiency of PrEP. In a focused uptake scenario where 25% of eligible MSM used PrEP for ten years, the number of person-years of PrEP use to avert one infection decreased to 97 person-years per HIV infection averted (95% SI: 88–108).

### Economic impact of pre-exposure prophylaxis implementation

**Figure 4:**
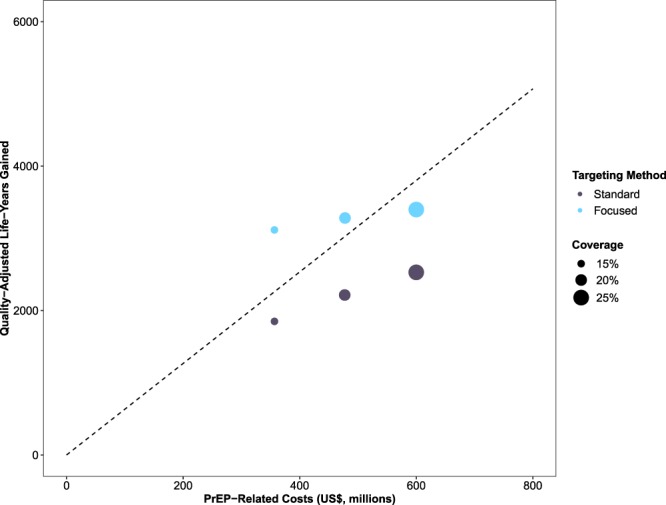
Incremental cost of pre-exposure prophylaxis (PrEP) implementation (*x*-axis) and the number of quality-adjusted life-years (QALYs) gained (*y*-axis) for each expansion scenario. Note: The dashed line represents the values for cost of PrEP implementation and the number of QALYs gained associated with an incremental cost-effectiveness ratio (ICER) of $100,000 per QALY gained. Scenarios above the line are deemed cost-effective based on this threshold.

Relative to no PrEP implementation, a standard uptake scenario where 15% of eligible MSM used PrEP for ten years generated an additional $356.6 billion in prevention-related costs (95% SI: $353.9–$359.7), but saved $106.9 billion in treatment-related costs (95% SI: $66.7–$144.6) by averting 317 new infections (95% SI: 198–429), resulting in a cost of $143,111 (95% SI: $85,090–$249,552) per QALY gained. Based on ICERs, no level of PrEP coverage in the standard uptake scenarios was considered cost-effective based on a threshold of $100,000 per QALY gained (Fig. 4).

Cost-effectiveness improved in focused uptake scenarios. A focused uptake scenario where 15% of eligible MSM used PrEP for ten years generated $356.6 billion in prevention-related costs (95% SI: $353.7–$359.5), but saved $180.1 billion in treatment-related costs (95% SI: $155.7–$201.9) by averting 534 new infections (95% SI: 462–599), resulting in a cost of $57,180 per QALY gained (95% SI: $44,135–$74,064) relative to a scenario without PrEP implementation and a savings of $57,802 per QALY gained (95% SI: $55,451–$60,158) relative to a standard uptake scenario with equivalent coverage. Based on ICERs, focused uptake scenarios where 15% and 20% of eligible MSM used PrEP for ten years were considered cost-effective relative to a scenario without PrEP implementation (Fig. 4) and cost-saving relative to standard uptake scenarios with equivalent coverage.

### Sensitivity analyses

In a sensitivity analyses in which male sex workers were 25% less likely to be adherent and retained on PrEP than other MSM, the number of new HIV infections increased by 13.1% (95% SI: -10.5–52.7%) in the standard uptake scenario where 25% of eligible MSM used PrEP for ten years. Similar increases were observed in the focused uptake scenario with equivalent coverage (34.6%; 95% SI: 10.3–60.5%). The cost per QALY gained increased by 90.0% (95% SI: 35.0–187.2%) in the standard uptake scenario and by 38.0% (95% SI: 9.2–78.5%) in the focused uptake scenario, reducing its cost-effectiveness.

## Discussion

Few studies have focused on PrEP implementation among cisgender male sex workers in the United States and, to date, no published studies have assessed the potential epidemiologic and economic impacts of focused expansion of PrEP use in this population. Rhode Island is a unique context for the study of HIV prevention among cisgender male sex workers. Between 1980 and 2009, prostitution was legal as there was no specific statute to define the act and outlaw it, although associated activities, such as solicitation, brothel-keeping, and procuring were illegal^25^. In addition, the capital city of Providence is home to Project Weber/RENEW, the only organization in the country dedicated exclusively to addressing the needs of male sex workers^15^. As such, there is an existing infrastructure to provide health and social services for this population, making the ambitious intervention coverage goals identified possible.

Our results suggest that focused expansion of PrEP use among cisgender male sex workers may be effective in reducing the overall number of new HIV infections in this low incidence setting in a manner that is cost-effective. By increasing the proportion of individuals on PrEP who are cisgender male sex workers from 5% (in a standard uptake scenario) to 25% (in a focused uptake scenario) while maintaining an overall coverage of 15% for ten years, an additional 217 HIV infections are averted in the overall population. Previous studies have referred to sex workers as a “core group”– a small population with a high number of sexual contacts that often has disproportionate role in sustaining transmission^26,27^. By averting HIV infections among cisgender male sex workers, further transmission events to their clients and other cisgender MSM are prevented, producing larger reductions in HIV incidence relative to other strategies. Previous research has shown that ensuring access to those most at risk for HIV infection can increase the impact of PrEP expansion in low incidence settings even in the context of expanding treatment access^28^.

However, despite these potential benefits, there are challenges in reaching these high levels of intervention coverage among cisgender male sex workers, with a death of evidence about how to best deliver PrEP to male sex workers. Nonetheless, recent studies have shown rapidly increasing interest in PrEP use among male sex workers^29,30^, suggesting that developing service delivery models that meet the needs of male sex workers should a public health priority. In a recent qualitative analysis with cisgender male sex workers in Rhode Island, Underhill and colleagues (2018) found that cyclical changes in risk among male sex workers responded to fluctuations in addiction severity, giving rise to a so-called “access-interest paradox”^3^. Much of male sex work practiced in Rhode Island is street-based and many individuals engage in sex work to meet survival and substance use needs^15^. During periods of intense substance use, individuals reported increased engagement in sex work and commensurate increases in interest using PrEP, but due to scarce resources and other barriers, many individuals were unable to access PrEP during these periods^3^. During times of reduced drug use and less frequent engagement in transactional sex, individuals reported greater access to resources to support PrEP initiation, but lower interest due to perceived lower risk for HIV infection^3^. Further, the results of our sensitivity analyses suggest that the impact of focused expansion of PrEP use among cisgender male sex workers may be diminished by reduced adherence and persistence. Our results, in combination with the existing literature^3–6^, suggest that additional outreach and financial support may be needed to support PrEP initiation, adherence, and persistence during these periods, including support in enrolling in health insurance, in context of interventions that reduce social and structural vulnerability such as the decriminalization of sex work^25^.

These analyses are subject to some limitations. First, the model does not account for the possibility that PrEP use may facilitate the emergence of drug resistance among those who initiate PrEP during acute stage HIV infection or those who acquire HIV infection with sub-therapeutic drug concentrations, although a previous economic evaluation found that potential emergent drug resistance does not impact estimates of effectiveness or cost-effectiveness of PrEP use at the population level^31^. Second, given that PrEP use has additional benefits associated with regular screening for sexually transmitted infections (STIs)^32^, our estimates may underestimate the true epidemiologic and economic impacts of PrEP implementation by focusing our analyses on costs saved due to averted HIV infections. Third, our findings are limited by key assumptions. These findings assume a population of male sex workers with elevated HIV prevalence and less frequent use of existing prevention strategies than other MSM. The impacts of targeted PrEP uptake among male sex workers may vary in settings with lower HIV prevalence or different distributions of behavioral risk factors among male sex workers, or in other legal or socio-structural contexts. Furthermore, we assumed stable PrEP coverage (such that scenarios could be directly compared), as opposed to increasing coverage over time, which has been observed in many settings. We also assumed no change in sexual risk behavior following periods of PrEP discontinuation. Given the lack of empirical data to guide an assumption, future modeling studies should examine the impact of increased sexual risk behavior after PrEP discontinuation on HIV transmission. Fourth, the model was parameterized to represent cisgender men only. As such, these findings cannot be readily generalized to transgender MSM. Fifth, local data were used to parameterize the model where possible, but as in many individual-based models, some input parameters were derived from different source populations, which may introduce bias and impact both the representativeness of the model and the generalizability of the simulation outputs^33^.

Targeted expansion of PrEP use among male sex workers may be a cost-effective strategy for reducing HIV incidence in this vulnerable population, their clients, and all other MSM. Strategies that prioritize male sex workers for PrEP initiation are considered cost-saving relative to those that do not. As such, interventions that reduce barriers to PrEP initiation and persistence among male sex workers are urgently needed.

## Supplementary information

Supplemental information

## Data Availability

The datasets generated during and/or analyzed during the current study are available from the corresponding author on reasonable request.
