# Supplementary material for: Potential Impact of Targeted HIV Pre-Exposure Prophylaxis Uptake Among Male Sex Workers
Source: Sci Rep. 2020 Mar 27;10:5650. doi: 10.1038/s41598-020-62694-5 (PMC7101419; doi:10.1038/s41598-020-62694-5)
Supplement: Supplementary file 1 — Supplemental information [file 41598_2020_62694_MOESM1_ESM.docx]

**Supplemental Appendix**

**Potential Impact of Targeted HIV Pre-Exposure Prophylaxis Uptake Among Male Sex Workers**

William C. Goedel, BA^1^

Matthew J. Mimiaga, ScD^,2,3^

Maximilian R.F. King, ScM^1^

Steven Safren, PhD^3,4^

Kenneth H. Mayer, MD^3,5,6^

Philip A. Chan, MD^7^

Brandon D.L. Marshall, PhD^1^

Katie Biello, PhD^1,2,3^

1. Department of Epidemiology, School of Public Health, Brown University, Providence, Rhode Island

2. Department of Behavioral and Social Sciences, School of Public Health, Brown University, Providence, Rhode Island

3. Fenway Institute, Fenway Health, Boston, Massachusetts

4. Department of Psychology, College of Arts and Sciences, University of Miami, Coral Gables, Florida

5. Division of Infectious Diseases, Beth Israel Deaconess Medical Center, Boston, Massachusetts

6. Department of Global Health and Population, T.H. Chan School of Public Health, Harvard University, Cambridge, Massachusetts

7. Department of Medicine, Warren Alpert Medical School, Brown University, Providence, Rhode Island

This supplemental appendix contains additional details regarding parameterization and calibration of the agent-based model described in this manuscript.

**About the TITAN Model**

The Treatment of Infectious Transmission in Agent-Based Networks (TITAN) model was created for the purpose of representing infectious disease transmission dynamics in networks of agents in diverse micro-epidemics within the United States and evaluating the impact of combination HIV prevention strategies on trajectories in HIV incidence and prevalence in these settings.^1^

The model includes only one type of entity: agents. Agents were characterized by several discrete state variables. All individuals are characterized by age, gender, race, sexual orientation, and HIV status. Agents with HIV infection are further characterized by their diagnosis status, their use of antiretroviral treatment, and their achievement of viral suppression. Agents without HIV infection are further characterized by their use of biomedical prevention services and their adherence to them. All discrete state variables, with the exception of gender, race, and sexual orientation, are dynamic and could be updated as time progressed. In the model, time is represented as a series of discrete time-steps each corresponding to one calendar month.

**Process Overview**

The model progresses through a series of routines in a given iteration of the model:

(1) *Creating a Population*: At the start of the simulation, model inputs are loaded, including the values of parameters governing the demographic, behavioral, and clinical characteristics of the individual agents. In addition, other technical details (e.g., the total number of iterations to be completed, the runtime allocated to complete the simulation) are loaded. The initial population of the model is created through a stochastic process that seeds the characteristics of agents according to the distributions of each of the parameters.

(2) *Creating a Network*: After creating the base population, the model begins by creating contact networks of agents through an algorithm that matches agents together. This partnering algorithm is based on pre-specified rules that govern the mixing of agents based on their demographic, behavioral, and clinical characteristics. The TITAN model is able to represent networks of sexual contact and/or injection-related contact in populations.

(3) *Simulating Interactions*: After creating the network, all agents interact with their partners. HIV transmission can occur through these interactions in serodiscordant pairs. In the event of a transmission event, an agent’s HIV status is updated from uninfected to infected.

(4) *Diagnosing and Treating Infections*: At the end of the partnering and interaction routines, agents can be tested for HIV infection and enter care if diagnosed. If diagnosed, agents can initiate antiretroviral treatment and achieve viral suppression. Agents who had been diagnosed with HIV infection at a previous time-step can also discontinue or re-initiate antiretroviral treatment during this process.

(5) *Summarizing Epidemiologic Outcomes*: After all processes have been completed, a number of key statistics are calculated at each time-step, including the number of new HIV infections and the number of newly diagnosed HIV infections within the population.

**Technical Details**

Python, an open-source programming language, was used for model coding, testing, and calibration. The simulations were run on Oscar, the primary research computing cluster located at the Brown University Center for Computation and Visualization. Oscar operates on the CentOS 6.7 Linux operating system and utilizes the Simple Linux Utility for Resource Management (SLURM) workload manager. The simulations were processed using Intel Xeon E5540 processors (2.53 gigahertz) operating with eight cores at 14.84 teraflops and 12 gigabytes of double date rate type III (DDR3) memory.

**Empirical Data Sources**

Most parameters governing the characteristics and behaviors of male sex workers are drawn from NEXUS, a recent longitudinal assessment of the social and sexual networks of a sample of male sex workers in New England (*n* = 95). Participants were recruited through community-based organization and outreach efforts in Massachusetts and Rhode Island between 2014 and 2016. Potential participants were eligible for study enrollment if they were aged 18 years or older, self-reported assignment of male sex at birth; self-reported their current gender identity as male; and self-reported engaging in anal intercourse with three or more men in exchange for money in the month prior to enrollment. Participants completed a network inventory, providing detailed information on each of their sexual partners in the preceding month.

Most parameters governing the characteristics and behaviors of other men who have sex with men (MSM) are drawn from estimates from the peer-reviewed literature (see “Model Processes” for further details). These parameters were described in detail in a previous publication.^2^ As such, we provide an overview of these parameters and their sources and focus on new features of this adaptation of the TITAN model.

**Model Processes**

Demographic Processes

The model is initialized in a virtual population of individuals representing the population of MSM aged 18 to 74 years old in Rhode Island. The overall population size was estimated based on two estimates published for Rhode Island. Using the Wide-Ranging Online Database for Epidemiologic Research (WONDER) maintained by the Centers for Disease Control and Prevention (CDC),^3^ we obtained number of adult men aged 15 to 74 years old in Rhode Island. First, applying a statistic generated by Grey and colleagues (2016) that estimated that 6.0% of adult men in Rhode Island were MSM,^4^ we generated a population size estimate of 23,519 MSM. Second, applying statistics generated by Lieb and colleagues (2011) that estimated that 6.8% of adult men in Rhode Island were MSM,^5^ we estimated a population size estimate of 26,517 MSM. Based on these two estimates, we assumed a final population size of 25,000.

The base population was seeded according to the age distribution for men in Rhode Island derived from WONDER (Table 1).^3^ The age distribution of male sex workers and other MSM were assumed to be similar.

**Table 1.** Age distribution and mortality rates of male population in Rhode Island

| **Age Group** | **Proportion** | **Mortality Rate** |
| --- | --- | --- |
| 15 to 24 years old | 20.2% | 0.64 |
| 25 to 34 years old | 17.4% | 1.39 |
| 35 to 44 years old | 15.9% | 2.11 |
| 45 to 54 years old | 19.1% | 4.71 |
| 55 to 74 years old | 27.3% | 12.82 |

Individuals exited the model in a deterministic fashion upon exceeding the age of 74 or through a stochastic process according to mortality rates that varied by age. Individuals who died or otherwise exited the population were replaced by an individual with characteristics drawn from the initial parameterized age distribution. These mortality rates were retrieved from the WONDER.^3^ Assuming that population growth is linear throughout the year, these proportions should represent reasonable estimates of instantaneous mortality rates (per 1,000 person-years) as they are based on mid-year population estimates.^6^ The mortality rates of male sex workers and other MSM were assumed to be similar (Table 1).

Both the stage of infection and stage of the continuum of care impact mortality among people living with HIV infection. Agents living with HIV infection who are using antiretroviral treatment and have achieved viral suppression are assumed to have comparable mortality rates to those who are not living with HIV infection.^7^ Among agents who are using antiretroviral treatment and have not achieved viral suppression, these mortality rates are increased by a factor of 3.^8^ In addition, these rates are increased by a factor of 10 for agents who have progressed to AIDS.^9^ Mortality was not assumed to differ among male sex workers.

The initial prevalence of HIV infection at model initialization is assumed to be 4.0% based on a recent estimate by Rosenberg and colleagues (2016).^10^ In the absence of an estimate specific to male sex workers in Rhode Island, we assumed an initial prevalence of 6.5%. This assumption is reflective of data from the National HIV Behavioral Surveillance System that showed that the prevalence of HIV infection is 1.5 times higher among men who report exchange sex relative to other MSM.^11^

Sexual Network and Behavior

During each time-step, agents determine their need for a partner and, if indicated, seek out and pair with other searching agents. A list of 100 partner-seeking agents that are able to mix with an index agent is enumerated and one of these agents is selected at random. This process is part of a negative binomial searching process in which partners are drawn from the population until each agent has achieved the necessary number of partners for the current time-step. Selection of a partner from this list is governed by both the age and sexual role of the index agent:

1. *Age Mixing*: The sexual networks among MSM are assortative by age.^12^ In general, agents will select sexual networks with partners who are close in age. Consistent with previous studies,^13^ this selection process is governed by the inverse of the absolute difference between the square roots of the ages of the two agents such that agents are more likely to pair with other agents who are close in age.

(2) *Sexual Role Mixing*: All agents are assigned a fixed sexual role preference (preferring the insertive role, preferring the receptive role, or preferring either role) drawn from the distribution below (Table 2).^14^ The model includes an absolute prohibition such that two exclusively insertive agents cannot partner nor can two exclusively receptive agents.

**Table 2.** Sexual role preferences among men who hae sex with men in Rhode Island

| **Sexual Role** | **Male Sex Workers** | **Other MSM** |
| --- | --- | --- |
| Insertive role only | 57.0% | 18.7% |
| Receptive role only | 26.0% | 10.8% |
| Versatile | 17.0% | 70.5% |

At model initialization, a value is drawn from a distribution representing the range of total numbers of male sexual partners per 12-month period. The value drawn from this distribution represents the mean value for an agent-specific distribution governing the index agent’s target number of sexual partners per year. At the beginning of each year, each index agent draws a new target number from this agent-specific distribution. This process allows for each agent to exhibit particular proclivities with regard to partner acquisition patterns from year-to-year without holding this behavior constant over time. The number of sexual partners per month (for male sex workers) and per year (for all other MSM) are shown in Table 3.^15^

**Table 3.** Number of sexual partners among men who have sex with men in Rhode Island

| **Number of Sexual Partners** | **Male Sex Workers** | **Other MSM** |
| --- | --- | --- |
| 0 partners | 19.0% | 8.0% |
| 1 partner | 8.0% | 24.0% |
| 2 partners | 6.0% | 17.0% |
| 3 to 4 partners | 21.0% | 18.0% |
| 5 to 9 partners | 24.0% | 25.0% |
| 10 partners or more | 22.0% | 8.0% |

At formation, each partnership is assigned a duration drawn from the distribution below (Table 4).^16^

**Table 4.** Relationship duration among men who have sex with men in Rhode Island

| **Partnership Duration** | **Male Sex Workers** | **Other MSM** |
| --- | --- | --- |
| Less than 1 month | 27.0% | 32.0% |
| 1 to 6 months | 22.0% | 26.0% |
| 7 to 12 months | 9.0% | 12.0% |
| 13 to 24 months | 9.0% | 12.0% |
| 26 to 36 months | 7.0% | 6.0% |
| 37 months or longer | 26.0% | 12.0% |

Similar to the process governing sexual partners, a value is drawn from a distribution representing the range of total number of condomless anal intercourse acts per partner. The value drawn from this distribution represents the mean value for the agent-specific distribution governing the agent’s target number of sex acts per partner in a given year. At the beginning of each year, agents draw a new target number from this agent-specific distribution, allowing each agent to exhibit proclivities with regard to sex frequency from year-to-year without holding these behaviors constant over time. The actual number of sex acts per month is the average of the target numbers for each agent in the partnership. The number of sexual acts per partner per month (for male sex workers) and per years (for all other MSM) are shown in Table 5.^16^

**Table 5.** Number of sex acts per partner for men who have sex with men in Rhode Island

| **Number of Sex Acts** | **Male Sex Workers** | **Other MSM** |
| --- | --- | --- |
| 1 act | 8.0% | 2.0% |
| 2 to 5 acts | 32.0% | 8.0% |
| 6 to 11 acts | 37.0% | 6.0% |
| 12 to 23 acts | 13.0% | 7.0% |
| 24 acts or more | 10.0% | 76.4% |

Condom-protected sexual acts are not explicitly simulated to increase computational efficiency.

HIV Transmission and Treatment

Parameters governing the per-act probability of HIV transmission are informed by a systematic review by Patel and colleagues (2014).^17^ In this adaptation of the model, only anal intercourse behaviors are simulated. The probability of HIV acquisition is 138 per 10,000 exposures for condomless insertive anal intercourse and 11 per 10,000 exposures for condomless receptive anal intercourse.^17^ To improve computational efficiency, only risk behaviors occurring within serodiscordant dyads are explicitly simulated. An individual’s total risk of HIV acquisition in a given time-step is dependent on the number of risk acts and whether the partner living with HIV infection is experiencing the acute stage of HIV infection, using antiretroviral treatment, or has achieved viral suppression.

Upon infection, agents experience a period of increased infectiousness lasting two time-steps that corresponds to the acute stage of HIV infection (where the base per-act probabilities of HIV infection are increased by a factor of 7.25).^17^ Following this period, individuals enter the chronic stage of HIV infection. All agents experience a base probability of progression to AIDS following infection (0.0029 per time-step).^18^ Both use of antiretroviral treatment and achievement of viral suppression slow progression to the late stage of HIV infection and, as such, these probabilities are decreased to 0.0021 per time-step for agents who are using antiretroviral treatment and have not achieved viral suppression and to 0.009 per time-step for agents who are using antiretroviral treatment and have achieved viral suppression.^18^

The proportions of people living with HIV infection who are diagnosed, on antiretroviral treatment, and with viral suppression are held constant for the duration of the simulation (see values for male sex workers and all other MSM below in Table 6).^19^ Both use of antiretroviral treatment and achievement of viral suppression by people living with HIV infection result in decreases in infectiousness. In dyads where the partner living with HIV infection is using antiretroviral treatment but has not achieved viral suppression, the base per-act probabilities of HIV infection are decreased by 19%.^20^ Individuals who have achieved viral suppression are assumed to have no risk of onward transmission to their sexual partners.^21,22^

**Table 6.** Care continuum metrics for men who have sex with men in Rhode Island

| **Care Continuum Stage** | **Male Sex Workers** | **Other MSM** |
| --- | --- | --- |
| Diagnosed with HIV infection | 83.0% | 89.0% |
| Antiretroviral treatment use | 78.0% | 79.0% |
| Viral load suppression | 47.0% | 50.6% |

At model initialization, it is assumed that 97.0% of male sex workers and 89.0% of all other MSM have ever been tested for HIV infection (Table 7).^15^ Agents who have ever been tested seek testing for HIV infection in any given time-step with a probability of 7.0% for male sex workers and 5.2% for all other MSM, assuming that 84.0% of male sex workers and 62.0% of all other MSM will test at least once in a given year (Table 7).^15^

**Table 7.** HIV testing practices among men who have sex with men in Rhode Island

| **HIV Testing** | **Male Sex Workers** | **Other MSM** |
| --- | --- | --- |
| Tested at least once | 97.0% | 89.0% |
| Tested at least once in past year | 84.0% | 62.0% |

Pre-Exposure Prophylaxis Use

The number of available prescriptions is determined based on the desired population coverage (see “Model Scenarios” for further detail), defined as the proportion of the population without HIV infection who use PrEP throughout the simulation window. In all scenarios, agents are eligible for PrEP if they (a) are in an ongoing relationship with an agent living with diagnosed HIV infection or (b) have two or more ongoing relationships and engage in condomless anal intercourse.^23^ In standard expansion scenarios, no further actions are taken to increase PrEP use among male sex workers and, as such, they remain equally likely as other MSM to initiate PrEP use. In targeted expansion scenarios, the overall coverage level is maintained but eligible male sex workers are five times more likely to initiated PrEP use than other MSM. The average number of individuals who use PrEP in a given month over time stratified by engagement in sex worker in standard and targeted expansion scenarios are shown in Supplemental Figure 1.

The impact of PrEP use on the probability of HIV infection is dependent on adherence. The efficacy associated with each level of adherence is derived from the STRAND trial, an open-label trial that aimed to established benchmarks for serum drug concentrations through directly observed dosing.^24^ In comparing those concentrations to those observed among individuals in the iPrEx trial, Anderson and colleagues (2012) concluded that the risk of HIV infection declined by 76% for individuals who took two doses per week, 96% for individuals who took four doses per week, and 99% for individuals who took seven doses per week.^24^ In line with these results,^24^ the per-act probability of HIV infection is reduced by 76% among agents in the model who take two to three pills per week and by 96% among agents who take four or more pills per week. Based on observed data from the PrEP program at the Rhode Island STD Clinic, it is assumed that 93.0% of patients achieve optimal levels of adherence.^2^ In the main analyses, adherence levels are assumed to be the same among male sex workers and other MSM.

The probability of discontinuation after initiation is modeled using a cumulative binomial distribution function calibrated to match the proportion who discontinue each quarter in the empirical data from the PrEP program at the Rhode Island STD Clinic (i.e., 72% are retained at 3 months post-initiation).^2^ In the main analyses, discontinuation rates are assumed to be the same among male sex workers and other MSM.

**Supplemental Figure 1.** Average number of individuals who use pre-exposure prophylaxis (PrEP) in a given month in each scenario, stratified by engagement in sex work


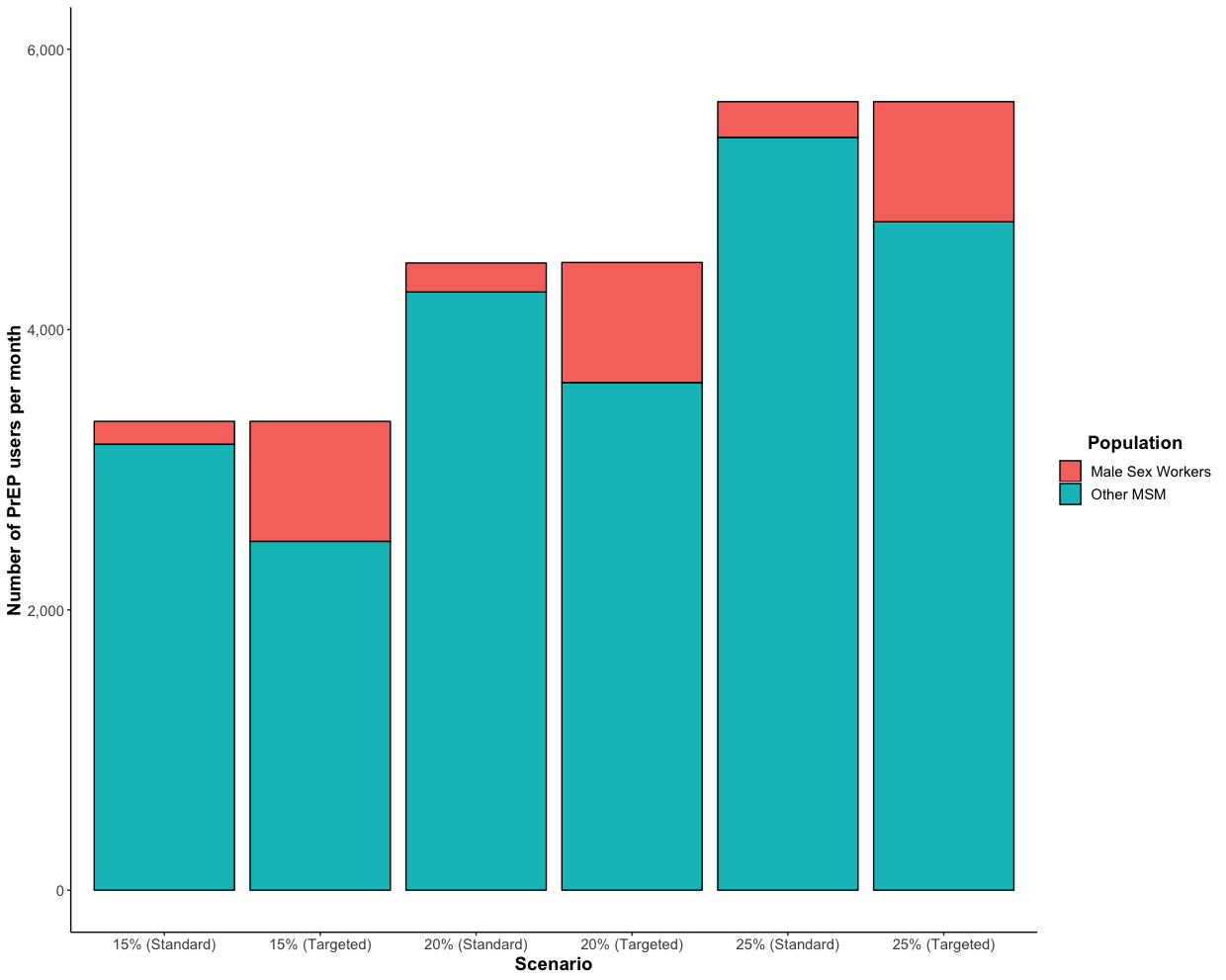


Cost and Utility Assumptions

The annual operating cost of providing PrEP to MSM living in Rhode Island were estimated using a health provider perspective. For HIV-uninfected individuals receiving PrEP in the model, patient monitoring costs were based on the current federal recommendations.^23^ These include HIV antibody testing and screening for STIs and monitoring serum creatinine levels every three months. Patient monitoring costs, including those associated with staff time, were derived from a cost analysis of the PrEP program at the Rhode Island STD Clinic.^25^ Previous cost-effectiveness modeling studies in the United States have used widely varying estimates of the annual cost of FTC/TDF for PrEP.^26-29^ Therefore, in our analyses, we used the median estimate of the annual cost of FTC/TDF for PrEP from the literature in our main analyses.^29^

These cost estimates were combined with the estimated NIA for each simulation to determine the average cost-utility of each PrEP implementation scenario in terms of the cost per quality-adjusted life-year (QALY) gained. The main cost-utility (R) formula was used:

$$R=\frac{C-(A\times T)}{(A\times Q)}$$

In this formula, “C” is the total cost associated with PrEP implementation, “A” is the NIA, “T” is lifetime treatment cost of one HIV infection, and “Q” is the number of QALYs saved by averting one HIV infection. The estimated discounted treatment cost of an HIV infection in 2012 United States dollars was $326,500.^30^ All costs were adjusted to 2015 United States dollars using the consumer price index for medical care.

Calculating the cost per QALY gained is the economic evaluation approach recommended by the United States Panel on Cost-Effectiveness in Health and Medicine.^31^ The number of QALYs gained by an intervention such as PrEP takes into account both increases in length of life and improvements in the perceived quality of life during surviving years. The number of QALYs gained by averting an HIV infection is estimated to be 5.83.^30^ All costs and QALYs described above are discounted at a rate of 3%.^31^

**Model Calibration**

The primary calibration target for the model was the successful recreation of the trends in the number of people living with diagnosed HIV infection (Supplemental Figure 1).^32^ Given that incident HIV infections emerge from characteristics and behaviors specified at the individual, the recreation of these epidemic behaviors provides some level of confidence that the model is able to represent essential components of the system. Model calibration was conducted using Latin hypercube sampling, a method developed to sample from and search a multidimensional parameter space.^33^ For each set of parameter values, the model was run and a statistic reflecting how closely the set recreates the empirical incidence rates was calculated. Parameter sets that did not provide adequate fit to these calibration targets were discarded to narrow the ranges from which values for input parameters can be sampled.

**Supplemental Figure 2.** Empirical (blue) and simulated (red) trends in number of gay, bisexual, and other men who have sex with men (MSM) living with diagnosed HIV infection in Rhode Island

**References**

1. Marshall BDL, Friedman SR, Monteiro JFG, et al. Prevention and treatment produced large decreases in HIV incidence in a model of people who inject drugs. *Health Aff (Millwood).* 2014;33(3):401-409.

2. Gantenberg JR, King MRF, Montgomery MC, et al. Improving the impact of HIV pre-exposure prophylaxis implementation in small urban centers among men who have sex with men: An agent-based modelling study. *PLoS One.* 2018;13(7):e0199915.

3. National Center for Health Statistics. Multiple Cause of Death File 2017. In. *Wide-Ranging Online Database for Epidemiologic Research*. Atlanta, Georgia: Centers for Disease Control and Prevention; 2018.

4. Grey JA, Bernstein KT, Sullivan PS, et al. Estimating the population sizes of men who have sex with men in US states and counties using data from the American Community Survey. *JMIR Public Health Surveill.* 2016;2(1):e14.

5. Lieb S, Fallon SJ, Friedman SR, et al. Statewide estimation of racial/ethnic populations of men who have sex with men in the U.S. *Public Health Rep.* 2011;126(1):60-72.

6. Vandenbroucke JP, Pearce N. Incidence rates in dynamic populations. *Int J Epidemiol.* 2012;41(5):1472-1479.

7. May MT, Gompels M, Delpech V, et al. Impact on life expectancy of HIV-1 positive individuals of CD4+ cell count and viral load response to antiretroviral therapy. *AIDS.* 2014;28(8):1193-1202.

8. Eyawo O, Franco-Villalobos C, Hull MW, et al. Changes in mortality rates and causes of death in a population-based cohort of persons living with and without HIV from 1996 to 2012. *BMC Infect Dis.* 2017;17(1):17.

9. Jensen-Fangel S, Pedersen L, Pedersen C, et al. Low mortality in HIV-infected patients starting highly active antiretroviral therapy: a comparison with the general population. *AIDS.* 2004;18(1):89-97.

10. Rosenberg ES, Grey JA, Sanchez TH, Sullivan PS. Rates of prevalent HIV infection, prevalent diagnoses, and new diagnoses among men who have sex with men in US states, metropolitan statistical areas, and counties, 2012-2013. *JMIR Public Health Surveill.* 2016;2(1):e22.

11. Nerlander LM, Hess KL, Sionean C, et al. Exchange sex and HIV infection among men who have sex with men: 20 US cities, 2011. *AIDS Behav.* 2017;21(8):2283-2294.

12. Grey JA, Rothenberg RB, Sullivan PS, Rosenberg ES. Disassortative age-mixing does not explain differences in HIV prevalence between young White and Black MSM: Findings from four studies. *PLoS One.* 2015;10(6):e0129877.

13. Jenness SM, Goodreau SM, Rosenberg ES, et al. Impact of the Centers for Disease Control's HIV preexposure prophylaxis guidelines for men who have sex with men in the United States. *J Infect Dis.* 2016;214(12):1800-1807.

14. Tieu HV, Li X, Donnell D, et al. Anal sex role segregation and versatility among men who have sex with men: EXPLORE study. *J Acquir Immune Defic Syndr.* 2013;64(1):121-125.

15. Chan PA, Rose J, Maher J, et al. A latent class analysis of risk factors for acquiring HIV among men who have sex with men: Implications for implementing pre-exposure prophylaxis. *AIDS Patient Care STDs.* 2015;29(11):597-605.

16. Wall KM, Stephenson R, Sullivan PS. Frequency of sexual activity with most recent male partner among young, Internet-using men who have sex with men in the United States. *J Homosex.* 2013;60(10):1520-1538.

17. Patel P, Borkowf CB, Brooks JT, Lasry A, Lansky A, Mermin J. Estimating per-act HIV transmission risk: A systematic review. *AIDS.* 2014;28(10):1509-1519.

18. Sterne JAC, Hernan MA, Ledergerber B, et al. Long-term effectiveness of potent antiretroviral therapy in preventing AIDS and death: A prospective cohort study. *Lancet.* 2005;366(9483):378-384.

19. State of Rhode Island. *Rhode Island Integrated Prevention and Care Comprehensive and Statewide Coordinated Statement of Need Plan.* Cranston, Rhode Island: Executive Office of Health and Human Services;2017.

20. Li Z, Purcell DW, Sansom SL, Hayes D, Hall HI. HIV transmission along the continuum of care - United States, 2016. *MMWR Morb Mortal Wkly Rep.* 2019;68(11):267-272.

21. Rodger AJ, Cambiano V, Bruun T, et al. Sexual activity without condoms and risk of HIV transmission in serodifferent couples when the HIV-positive partner is using suppressive antiretroviral therapy. *JAMA.* 2016;316(2):171-181.

22. Rodger AJ, Cambiano V, Bruun T, et al. Risk of HIV transmission through condomless sex in serodifferent gay couples with the HIV-positive partner taking suppressive antiretroviral therapy (PARTNER): Final results of a multicentre, prospective, observational study. *Lancet.* 2019.

23. United States Public Health Service. *Pre-Exposure Prophylaxis for the Prevention of HIV Infection in the United States - 2017 Updated.* Atlanta, Georgia: Centers for Disease Control and Prevention;2017.

24. Anderson PL, Glidden DV, Liu AY, et al. Emtricitabine-tenofovir concentrations and pre-exposure prophylaxis efficacy in men who have sex with men. *Sci Transl Med.* 2012;4(151):151ra125.

25. Younus A. *Cost analysis of a pre-exposure prophylaxis (PrEP) program for HIV prevention in Rhode Island: A micro-costing approach*. Providence, Rhode Island: School of Public Health, Brown University; 2017.

26. Desai K, Sansom SL, Ackers ML, et al. Modeling the impact of HIV chemoprophylaxis strategies among men who have sex with men in the United States: HIV infections prevented and cost-effectiveness. *AIDS.* 2008;22(14):1829-1839.

27. Koppenhaver RK, Sorensen SW, Farnham PG, Sansom SL. The cost-effectiveness of pre-exposure prophylaxis in men who have sex with men in the United States: An epidemic model. *J Acquir Immune Defic Syndr.* 2011;58(2):e51-e52.

28. Juusola JL, Brandeau ML, Owens DK, Bendavid E. The cost-effectiveness of preexposure prophylaxis for HIV prevention in the United States in men who have sex with men. *Ann Intern Med.* 2012;156(8):541-550.

29. Chen A, Dowdy DW. Clinical effectiveness and cost-effectiveness of HIV pre-exposure prophylaxis in men who have sex with men: Risk calculators for real-world decision-making. *PLoS One.* 2014;9(10):e108742.

30. Farnham PG, Holtgrave DR, Gopalappa C, Hutchinson AB, Sansom SL. Lifetime costs and quality-adjusted life years saved from HIV prevention in the test and treat era. *J Acquir Immune Defic Syndr.* 2013;64(2):e15-e18.

31. Sanders GD, Neumann PJ, Basu A, et al. Recommendations for conduct, methodological practices, and reporting of cost-effectiveness analyses: Second Panel on Cost-Effectiveness in Health and Medicine. *JAMA.* 2016;316(10):1093-1103.

32. Atlas Plus. National Center for HIV/AIDS, Viral Hepatitis, Sexually Transmitted Diseases, and Tuberculosis Prevention; 2017. <https://www.cdc.gov/nchhstp/atlas/index.htm>.

33. McKay MD, Beckman RJ, Conover WJ. A comparison of three methods for selecting values of input variables in the analysis of output from a computer code. *Technometrics.* 1979;21(2):239-245.
